# Supplementary material for: Huntingtin structure is orchestrated by HAP40 and shows a polyglutamine expansion-specific interaction with exon 1
Source: Commun Biol. 2021 Dec 8;4:1374. doi: 10.1038/s42003-021-02895-4 (PMC8654980; doi:10.1038/s42003-021-02895-4)
Supplement: Supplementary file 2 — Supplementary Information [file 42003_2021_2895_MOESM2_ESM.pdf]

Huntingtin structure is orchestrated by HAP40 and shows a polyglutamine expansion-specific interaction with exon 1

Rachel J. Harding*[1], Justin Deme[2,3,4], Johannes F. Hevler[5,6], Sem Tamara[5,6], Alexander Lemak[7], Jeffrey P. Cantle[8], Magdalena M. Szewczyk[1], Nola Begeja[9], Siobhan Goss[9], Xiaobing Zuo[10], Peter Loppnau[1], Alma Seitova[1], Ashley Hutchinson[1], Lixin Fan[11], Ray Truant[9], Matthieu Schapira[1,12], Jeffrey B. Carroll[8], Albert J. R. Heck[5,6], Susan M. Lea[2,3,4], Cheryl H. Arrowsmith*[1,7]

[1] Structural Genomics Consortium, University of Toronto, Ontario, M5G 1L7, Canada
[2] Sir William Dunn School of Pathology, University of Oxford, South Parks Road, Oxford, OX1 3RE, UK
[3] Central Oxford Structural Molecular Imaging Centre, University of Oxford, South Parks Road, Oxford, OX1 3RE, UK
[4] Center for Structural Biology, Center for Cancer Research, National Cancer Institute, Frederick, Maryland, 21702, USA
[5] Biomolecular Mass Spectrometry and Proteomics, Bijvoet Center for Biomolecular Research and Utrecht Institute of Pharmaceutical Sciences, Utrecht University, Padualaan 8, 3584 CH Utrecht, The Netherlands
[6] Netherlands Proteomics Center, Padualaan 8, 3584 CH Utrecht, The Netherlands
[7] Princess Margaret Cancer Centre and Department of Medical Biophysics, University of Toronto, Toronto, Ontario, M5G 1L7, Canada
[8] Behavioral Neuroscience Program, Department of Psychology, Western Washington University, Bellingham, Washington, 98225, USA
[9] Department of Biochemistry and Biomedical Sciences, McMaster University, Hamilton, Ontario, L8S 4K1, Canada
[10] X-ray Science Division, Argonne National Laboratory, Lemont, Illinois, 60439, USA
[11] Basic Science Program, Frederick National Laboratory for Cancer Research, SAXS Core of NCI, National Institutes of Health, Frederick, Maryland, 21701, USA
[12] Department of Pharmacology & Toxicology, University of Toronto, Toronto, Ontario, M5S 1A8, Canada

*Corresponding authors:

Rachel J. Harding (0000-0002-1134-391X) Rachel.Harding@utoronto.ca
Cheryl H. Arrowsmith (0000-0002-4971-3250) Cheryl.Arrowsmith@uhnresearch.ca

Supplementary Table 1. Residues contributing to putative ligand-able pocket at interface of HTT and HAP40

	Pocket Contributing Residues
HTT	L1015, R1021, T1024, M1071, T1074, L1075, S1078, W1080
HAP40	P84, A87, L88, T91, E92, R95, H132, Q137, A139, A140, A143, L144, L146, E147, A150, R153, F165, E186

Supplementary Table 2. Experimental cross-links involving exon1 residues observed for HTT-HAP40 Q54

For the purposes of the modelling experiments, HAP40 lysine residues are listed with numbering which continue on from the HTT sequence, the true numbering of the residues for HAP40 are in brackets.

Cross-link ID	Lysine Position 1	Region	Lysine Position 2	Region
1	6	Exon1	9	Exon1
2	6	Exon1	92	N-HEAT
3	9	Exon1	92	N-HEAT
4	6	Exon1	98	N-HEAT
5	9	Exon1	98	N-HEAT
6	6	Exon1	99	N-HEAT
7	6	Exon1	158	N-HEAT
8	6	Exon1	220	N-HEAT
9	6	Exon1	262	N-HEAT
10	6	Exon1	337	N-HEAT
11	6	Exon1	444	IDR
12	6	Exon1	1190	N-HEAT
13	6	Exon1	1203	N-HEAT
14	6	Exon1	1204	N-HEAT
15	9	Exon1	1204	N-HEAT
16	6	Exon1	1404	N-HEAT
17	6	Exon1	1417	N-HEAT
18	6	Exon1	1436	N-HEAT
19	6	Exon1	1559	N-HEAT
20	6	Exon1	1564	N-HEAT
21	6	Exon1	2536	C-HEAT
22	6	Exon1	2548	C-HEAT
23	6	Exon1	2759	C-HEAT
24	6	Exon1	3206 (32)	HAP40
25	9	Exon1	3206 (32)	HAP40
26	6	Exon1	3214 (40)	HAP40

Supplementary Table 3. Experimental cross-links involving exon1 residues observed for HTT-HAP40 Q23

For the purposes of the modelling experiments, HAP40 lysine residues are listed with numbering which continue on from the HTT sequence, the true numbering of the residues for HAP40 are in brackets.

Cross-link ID	Lysine Position 1	Region	Lysine Position 2	Region
1	6	Exon1	92	N-HEAT
2	6	Exon1	98	N-HEAT
3	9	Exon1	92	N-HEAT
4	9	Exon1	98	N-HEAT
5	6	Exon1	99	N-HEAT
6	9	Exon1	99	N-HEAT
7	6	Exon1	1190	N-HEAT
8	6	Exon1	1203	N-HEAT
9	6	Exon1	1204	N-HEAT
10	9	Exon1	1204	N-HEAT
11	6	Exon1	1417	N-HEAT
12	6	Exon1	3206 (32)	HAP40
13	6	Exon1	3214 (40)	HAP40

Supplementary Table 4. Experimental cross-links involving IDR residues observed for HTT-HAP40 Q23

Cross-link ID	Lysine Position 1	Region	Lysine Position 2	Region
1	444	IDR	220	N-HEAT
2	648	IDR	220	N-HEAT
3	444	IDR	262	N-HEAT
4	664	IDR	669	N-HEAT
5	648	IDR	826	N-HEAT
6	444	IDR	633	IDR
7	633	IDR	648	IDR
8	633	IDR	664	IDR
9	648	IDR	664	IDR

Supplementary Table 5. Experimental cross-links involving IDR residues observed for HTT-HAP40 Q54

Cross-link ID	Lysine Position 1	Region	Lysine Position 2	Region
1	475	IDR	6	Exon1
2	475	IDR	123	N-HEAT
3	475	IDR	475	N-HEAT
4	664	IDR	251	N-HEAT
5	475	IDR	293	N-HEAT
6	475	IDR	368	N-HEAT
7	679	IDR	700	N-HEAT
8	695	IDR	700	N-HEAT
9	664	IDR	679	IDR

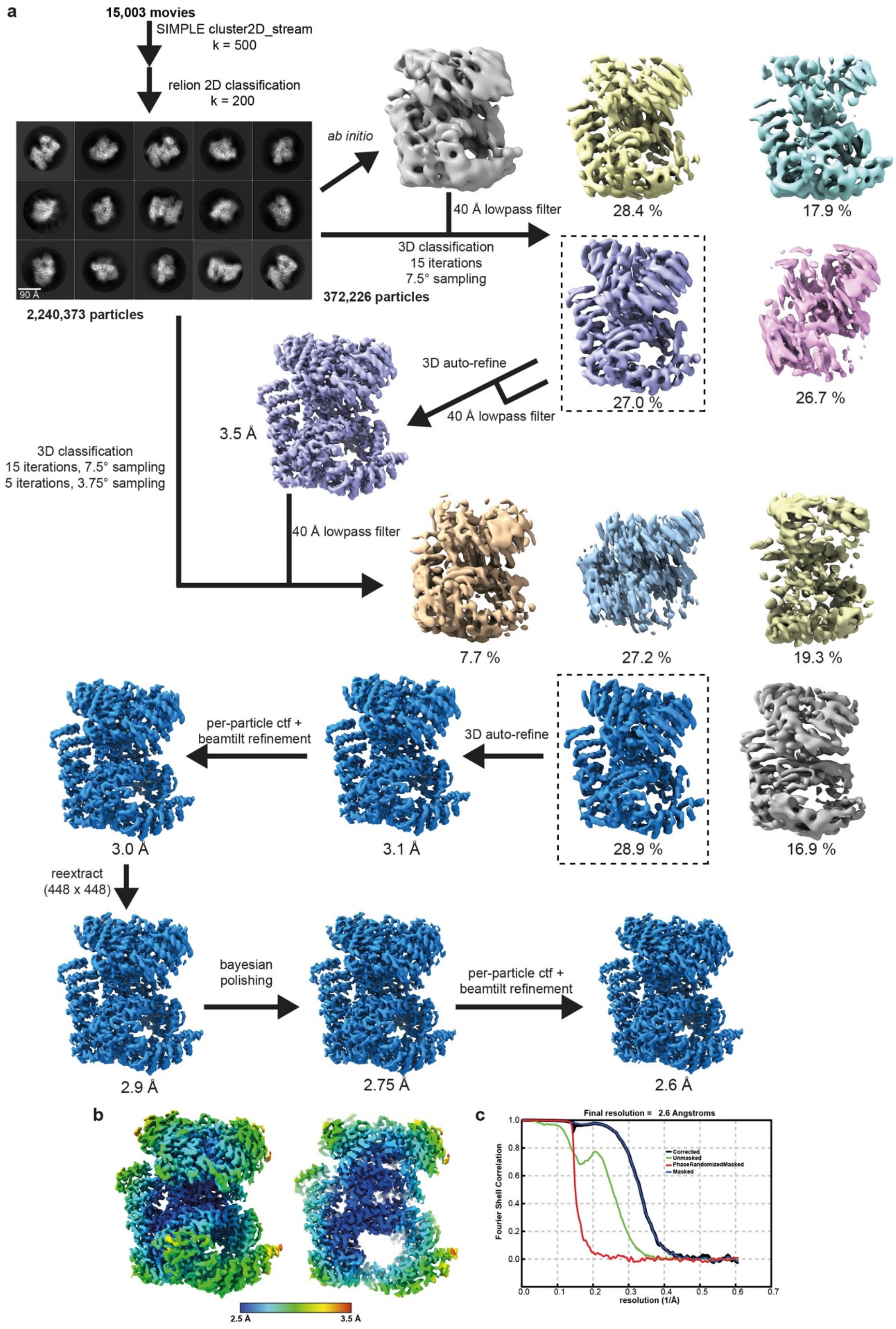

Supplementary Figure 1. Cryo-EM processing workflow and local/global map quality for HTT-HAP40.
a Image processing workflow for HTT-HAP40. **b** Local resolution estimation of reconstructed map as determined within RELION. Left, full map; right, central slab through map. **c** Gold-standard Fourier Shell Correlation (FSC) plot used for global resolution estimation as determined within RELION.

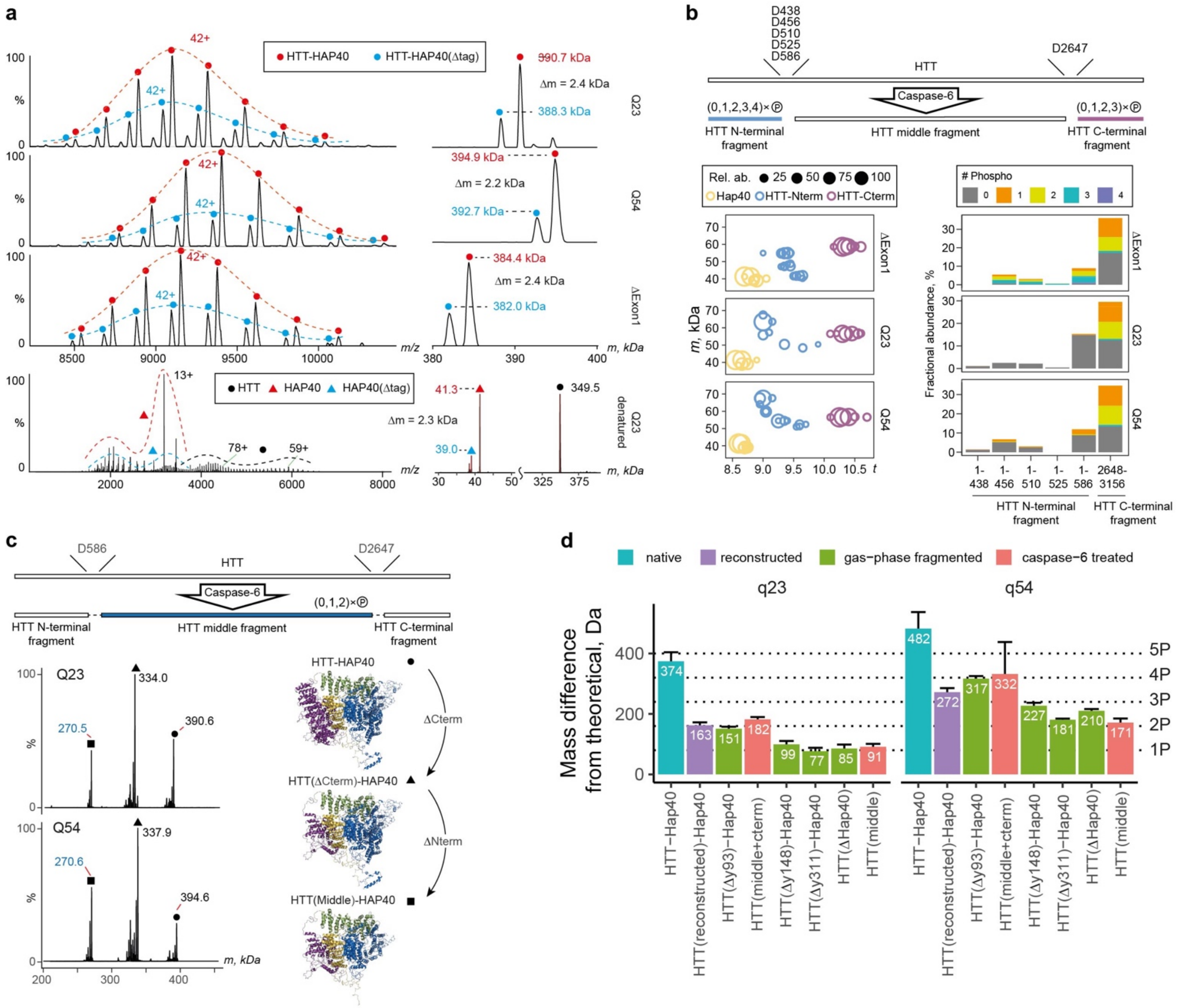

Supplementary Figure 2. Determining the different proteoforms of HTT using hybrid mass spectrometry approach.

a Native mass spectra for HTT-HAP40 Q23, Q54 and Δexon 1 samples. Top three left panels: annotated native raw spectra of HTT-HAP40 Q23, Q54 and Δexon 1. Top three right panels: annotated mass distributions of HTT-HAP40 Q23, Q54 and Δexon 1. Bottom panel: denaturing MS of HTT-HAP40 Q23 and respective mass distribution. Denaturing MS assigns the second peak, which is ~2.4 kDa smaller than the main peak and corresponds to the expected mass of the complex with the N-terminal His-tag cleaved from HAP40. **b** Middle-down MS of Caspase6-treated HTT-HAP40 complexes. Top panel: schematics of HTT digestion using Caspase6. Bottom left panel: mass-feature maps for digested and denatured HTT-HAP40 samples. Bottom right panel: abundances of N-terminal and C-terminal HTT proteoforms with 0-4 phosphorylation motifs (assigned by mass). **c** Native MS of Caspase6-treated HTT-HAP40 Q23 and Q54 complexes. Top panel: schematics of HTT digestion using Caspase6 with the uncleaved HTT-middle region highlighted. Bottom left panel: mass distributions of Caspase6-digested and gas-phase-activated HTT-HAP40. Bottom right panel: structures of HTT-HAP40 for the major species observed in native spectra on the left. **d** Difference between the theoretical and experimental masses of HTT-HAP40 complex samples under various conditions (native, Caspase6-treated and denatured, and gas-phase fragmented). Reconstructed masses were obtained by summing masses of HTT-middle determined by native MS and intensity-weighted average masses of HTT C-terminal and HTT N-terminal fragments produced upon Caspase6 treatment and determined in middle-down MS. Error bars represent mean error of mass determination. Together, this analysis indicates approximately two phosphorylation modifications carried by HTT Q23 on average (additional 163 Da mass, purple).

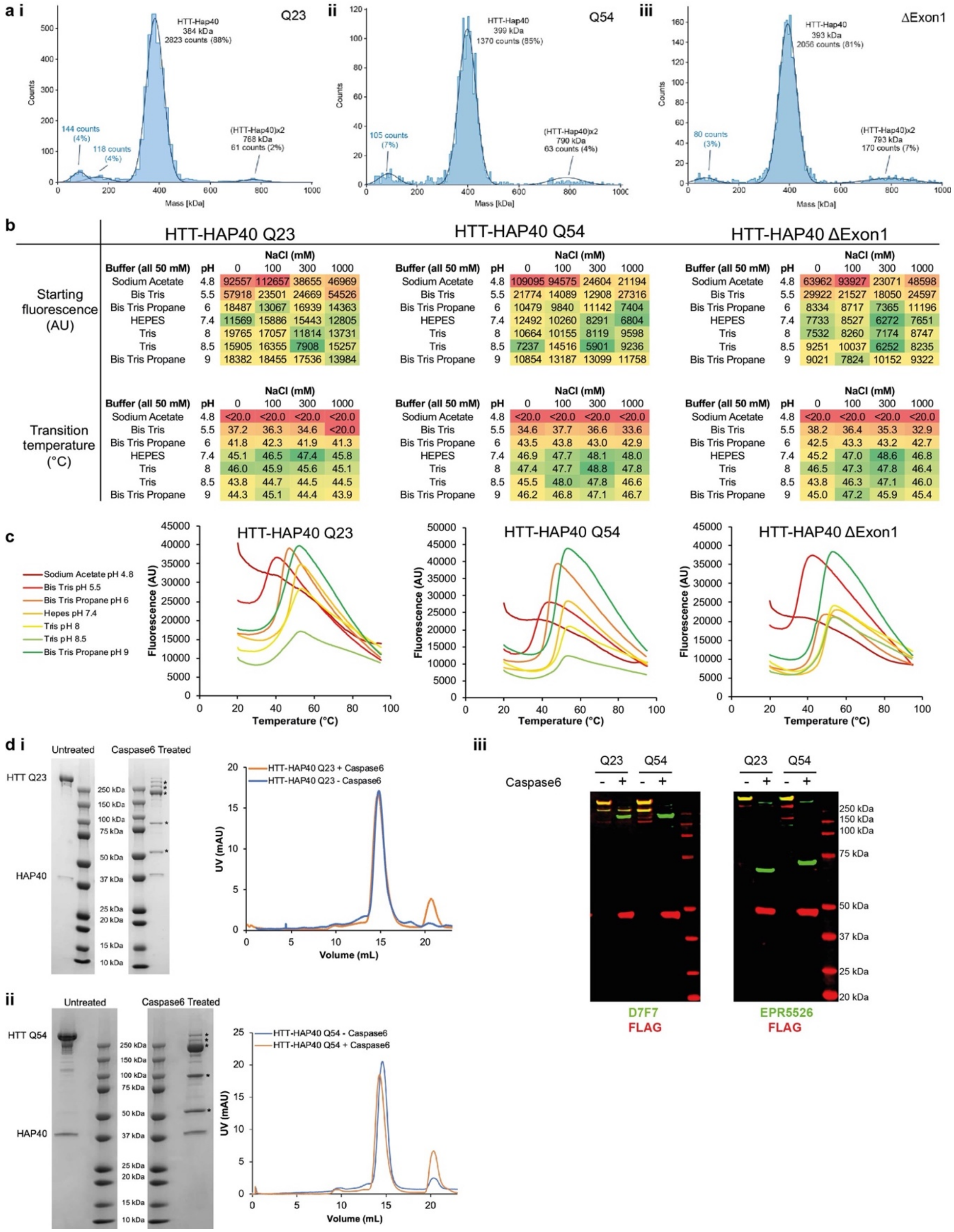

Supplementary Figure 3. Structural stability and monodispersity of HTT-HAP40 complexes probed with buffer screening and proteolytic cleavage.

a Mass photometry analysis of HTT-HAP40 Q23, Q54 and Δexon 1. **b** Assessing complex stability by measuring transition temperature using DSF in a range of different buffer and salt conditions. **c** DSF profiles of HTT-HAP40 samples in different buffer conditions with 300 mM NaCl. **d** Caspase6 cleavage of **i** Q23 and **ii** Q54 HTT-HAP40 proteins into smaller HTT fragments (*) assessed by SDS-PAGE (left), analytical gel filtration (right) and **iii** western blot with antibodies recognising before (EPR5526) and after (D7F7) the documented Caspase6 cleavage site (D586) as well as anti-FLAG which recognises the C-terminal FLAG-tag of the samples.

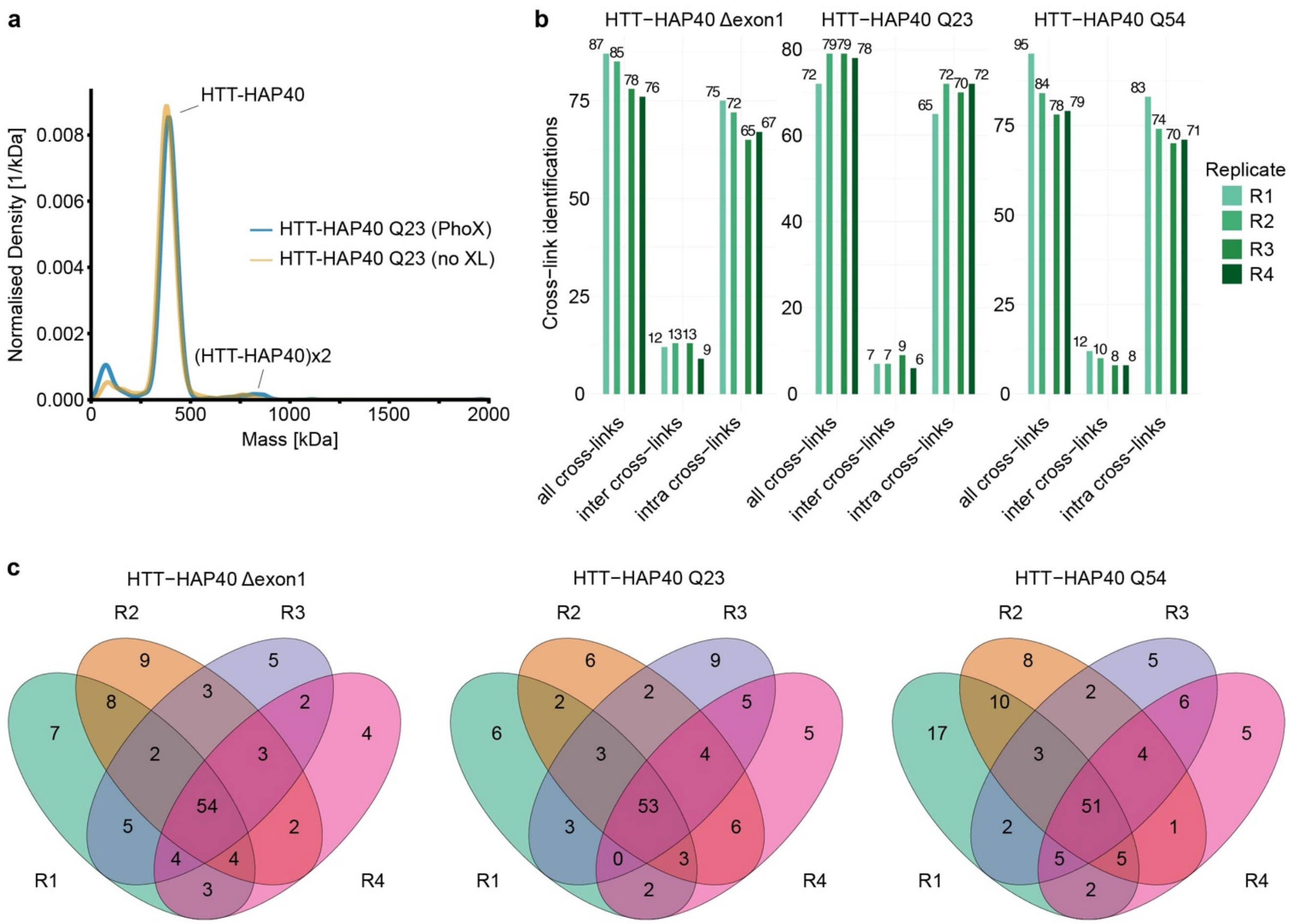

Supplementary Figure 4. Mass photometry and validation of cross-linking mass spectrometry analysis of HTT-HAP40.

a Mass photometry analysis of HTTQ23-HAP40 untreated (no XL) or treated (PhoX) indicates no artificial HTT-HAP40 oligomers or conformers are introduced by cross-linking. **b** The number of total cross-links, intra-links (HTT-HTT or HAP40-HAP40) and inter-links (HTT-HAP40) observed for each replicate and sample. Similar cross-link identifications are found across replicates and samples highlighting the reproducibility of our data. **c** Venn diagram for cross-linking mass spectrometry reproducibility. For HTT Δexon1-HAP40, 78% of the cross-links are observed in 2 out of 4 replicates. For HTT Q23-HAP40 76% of cross-links are observed in 2 out of 4. For HTT Q54-HAP40 72 % of the cross-links are observed in 2 out of 4 replicates.

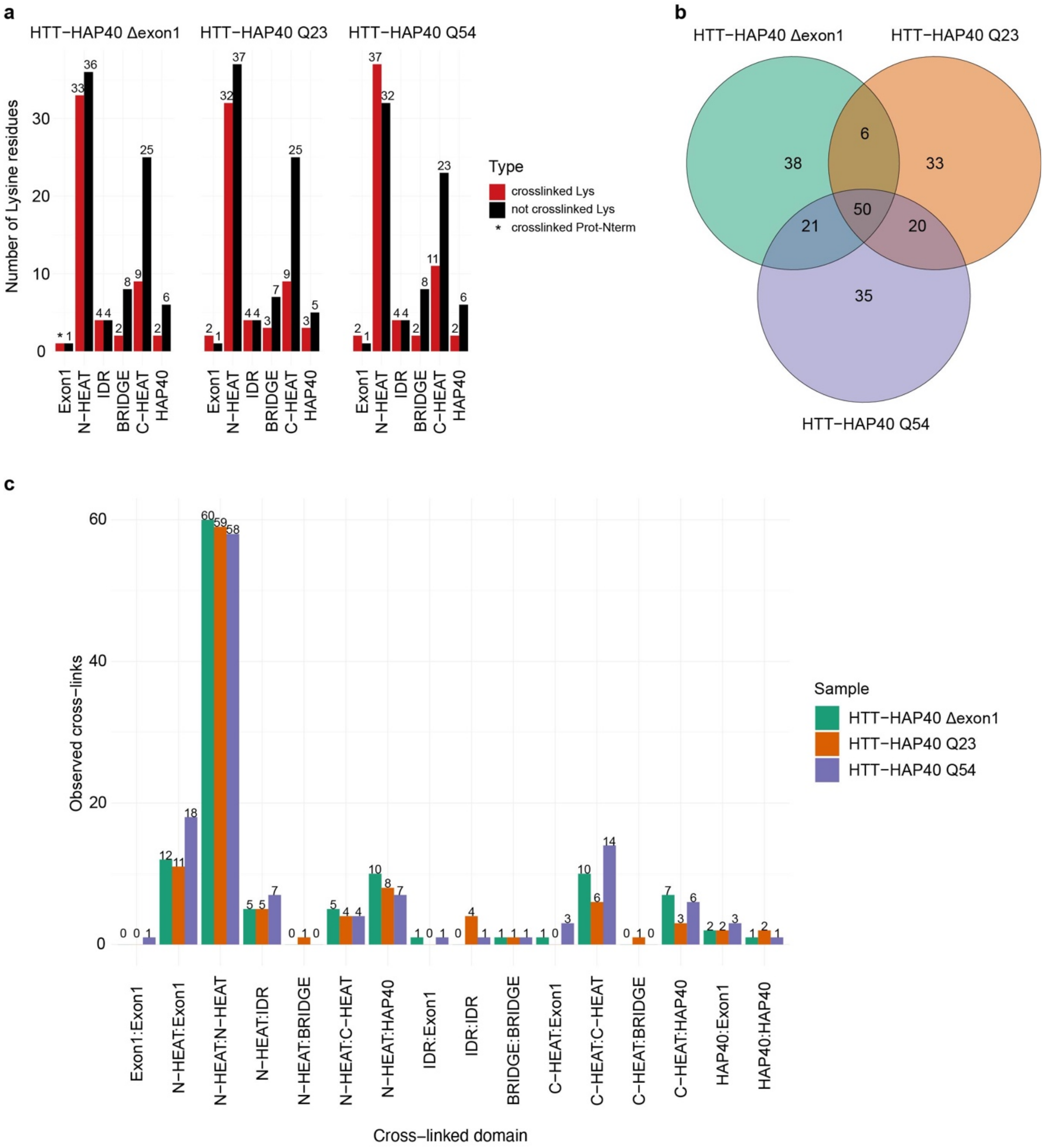

Supplementary Figure 5. Analysis and validation of identified HTT-HAP40 cross-links.

a Overview of cross-linked and unmodified lysine residues for each functional domain. For all available domains in each sample cross-link sites are identified indicating good cross-linking efficiency. For all domains, excluding the C-HEAT and HAP40, ~50% of available lysine residues are cross-linked. Lysine residues in the C-HEAT and HAP40 are less accessible for the cross-linking which is supported by our cryoEM model which shows many are buried and inaccessible for chemical modification compared to other regions such as the N-HEAT domain. **b** Venn diagram for reproducibility between different HTT-HAP40 variant protein samples. A high degree of overlap between different variants supports the conclusion that structural differences resulting from polyglutamine expansion or deletion of exon1 are not extreme and the general structure and conformation of the complex is retained in all three samples. **c** Overview of cross-linked domains across different HTT-HAP40 samples indicating similar cross-links are identified for all samples. Three cross-links are mapped for exon1 to a specific surface area of the C-HEAT domain for HTT-HAP40 Q54. The data supporting these cross-links is highly robust (XL1: Pos 2790 – Pos 6 (3 CSMs), XL2: Pos 2579 – Pos 6 (4 CSMs), XL3: Pos 2567 – Pos 6 (1 CSMs)) and the residues are structurally close to each other indicating this is a reproducible conformation of the expanded form of exon1 not seen for the wildtype exon1.

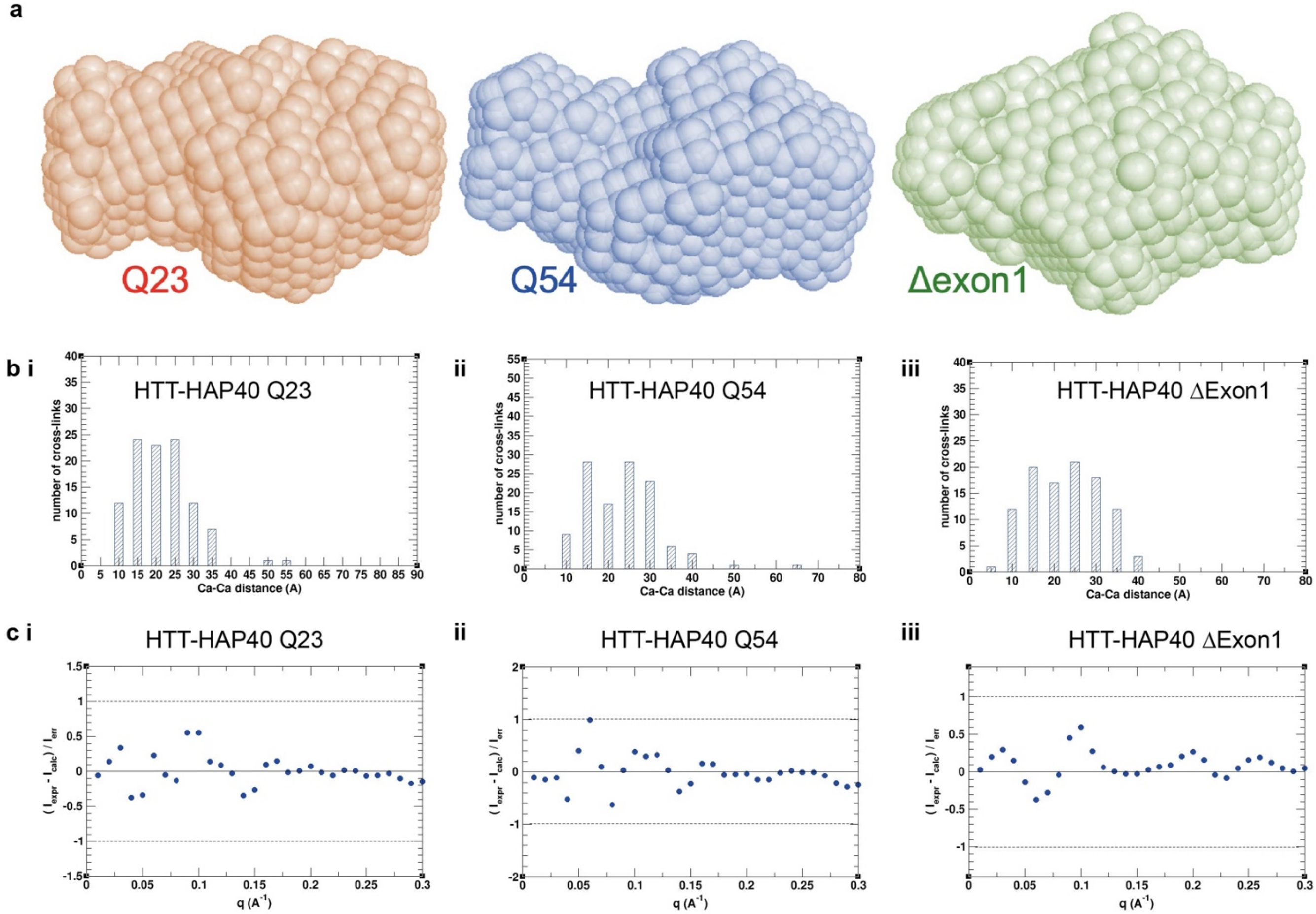

Supplementary Figure 6. SAXS and cross-linking mass spectrometry analysis of HTT-HAP40.

a SAXS envelopes calculated for HTT-HAP40 Q23 (red), Q54 (blue) and Δexon 1 (green). **b** Consistency of the experimental cross-links with an ensemble of models. Each histogram bar shows number of cross-links that have the corresponding Cα-Cα distances (the minimal distance over all models in the ensemble) falling within the corresponding distance bin. The histogram bars corresponding to the distances of > 35 Å indicate the number of cross-links that are inconsistent with the ensemble. **c** Normalized difference between the experimental SAXS profile and the theoretical profiles averaged over the ensemble.

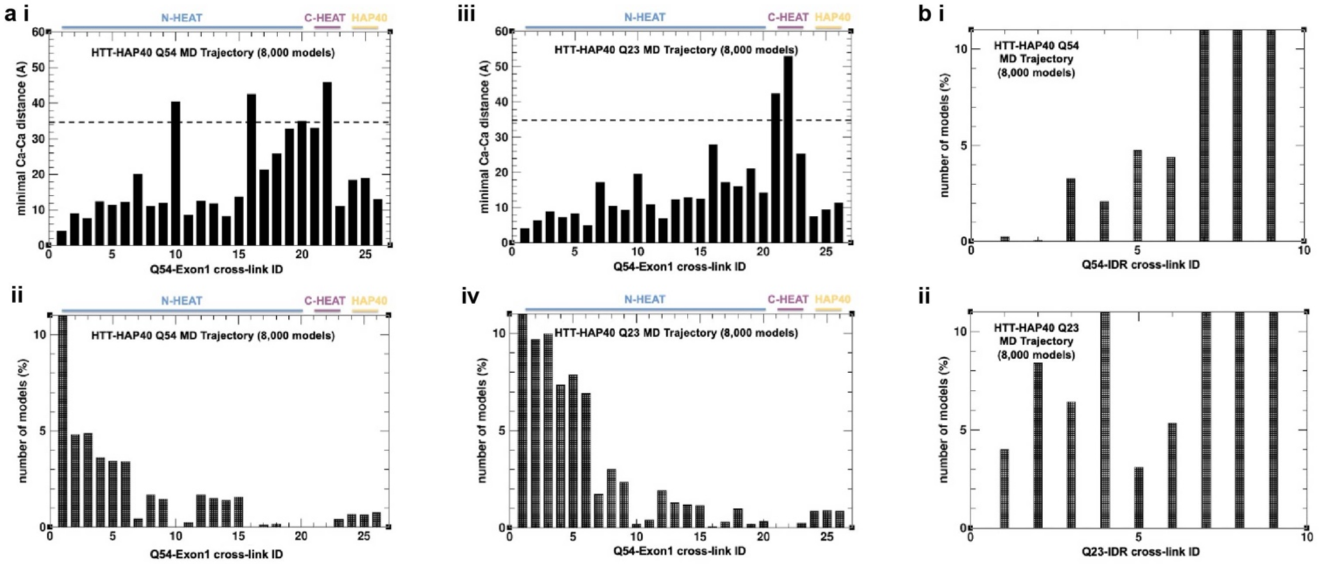

Supplementary Figure 7. Validation of polyglutamine dependent structural changes to HTT-HAP40. **a** Statistics of cross-links experimentally observed for HTT-HAP40 Q54 complex (numbered as per **Supplementary Table 3**) in two molecular dynamics ensembles calculated for HTT-HAP40 Q54 (**i**, **ii**) and HTT-HAP40 Q23 (**iii**, **iv**) complexes, respectively. Results for 26 cross-links that involve exon 1 N-terminal residues (K6 and K9) are shown. **i** and **iii** A cross-link Ca-Ca distance minimal in the ensemble consisting of 8,000 models obtained by unconstrained MD simulations. We assume that a cross-link could be formed when the corresponding Ca-Ca distance is < 35 Å. **ii** and **iv** Percentage of the models in the ensemble that have two Lys residues close enough to form a cross-link. **b** Statistics of cross-links experimentally observed for HTT-HAP40 Q23 (**i**) and HTT-HAP40 Q54 (**ii**) complexes that involve IDR residues of HTT (numbered as per **Supplementary Tables 5** and **6**). Percentage of the models in MD ensemble that have two Lys residues close enough to form a cross-link are shown for the ensembles of HTT-HAP40 Q23 (**i**) and HTT-HAP40 Q54 (**ii**) that were obtained by unconstrained MD simulations.

a

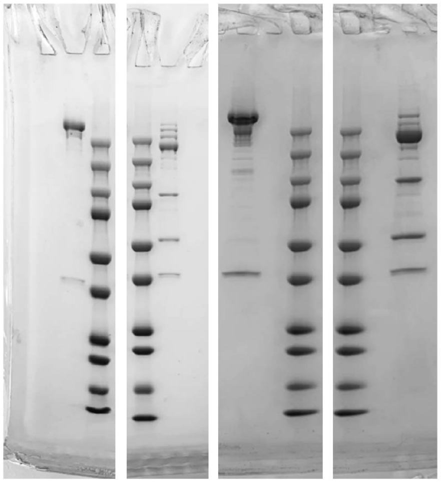

b

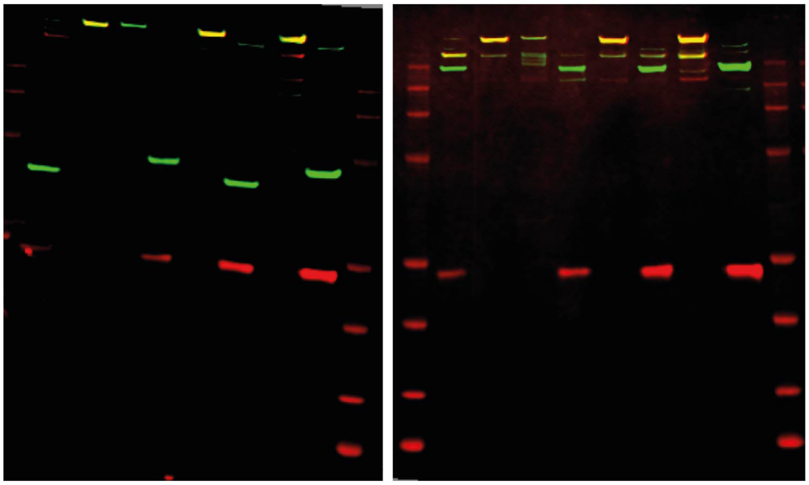

Supplementary Figure 8 - Uncropped gel and blot images

a SDS-PAGE shown in Figure 4 and Supplementary Figure 3. **b** Western blot show in Supplementary Figure 3.
