# Supplementary material for: Huntingtin structure is orchestrated by HAP40 and shows a polyglutamine expansion-specific interaction with exon 1
Source: Commun Biol. 2021 Dec 8;4:1374. doi: 10.1038/s42003-021-02895-4 (PMC8654980; doi:10.1038/s42003-021-02895-4)
Supplement: Supplementary file 3 — Description of Additional Supplementary Files [file 42003_2021_2895_MOESM3_ESM.pdf]

Description of Additional Supplementary Files

File name: Supplementary Movie 1
Description: Exon 1 of huntingtin (HTT) occupies different conformational space in the wildtype form of the HTT-HAP40 complex compared to Huntington's disease form

File name: Supplementary Data 1
Description: Multiple sequence alignment for HTT used for Consurf analysis

File name: Supplementary Data 2
Description: Multiple sequence alignment for HAP40 used for Consurf analysis

File name: Supplementary Data 3
Description: Apo HTT cryo-EM map

File name: Supplementary Data 4
Description: HTT-HAP40 Q23 regularised SAXS profile

File name: Supplementary Data 5
Description: HTT-HAP40 Q54 regularised SAXS profile

File name: Supplementary Data 6
Description: HTT-HAP40 Δexon 1 regularised SAXS profile 25

File name: Supplementary Data 7
Description: XL-MS data

File name: Supplementary Data 8
Description: HTT-HAP40 ensemble weightings

File name: Supplementary Data 9
Description: HTT-HAP40 Q23 ensemble models

File name: Supplementary Data 10
Description: HTT-HAP40 Q54 ensemble models

File name: Supplementary Data 11
Description: HTT-HAP40 Δexon 1 ensemble models
