# Supplementary material for: Huntingtin structure is orchestrated by HAP40 and shows a polyglutamine expansion-specific interaction with exon 1
Source: Commun Biol. 2021 Dec 8;4:1374. doi: 10.1038/s42003-021-02895-4 (PMC8654980; doi:10.1038/s42003-021-02895-4)
Supplement: Supplementary file 16 — Reporting Summary [file 42003_2021_2895_MOESM16_ESM.pdf]

nature portfolio

Corresponding author(s): Harding, Rachel, Arrowsmith, Cheryl

Last updated by author(s): 2021/11/02

Reporting Summary

Nature Portfolio wishes to improve the reproducibility of the work that we publish. This form provides structure for consistency and transparency in reporting. For further information on Nature Portfolio policies, see our Editorial Policies and the Editorial Policy Checklist.

Statistics

For all statistical analyses, confirm that the following items are present in the figure legend, table legend, main text, or Methods section.

n/a	Confirmed	
☐	☒	The exact sample size (*n*) for each experimental group/condition, given as a discrete number and unit of measurement
☐	☒	A statement on whether measurements were taken from distinct samples or whether the same sample was measured repeatedly
☐	☒	The statistical test(s) used AND whether they are one- or two-sided<br>*Only common tests should be described solely by name; describe more complex techniques in the Methods section.*
☒	☐	A description of all covariates tested
☐	☒	A description of any assumptions or corrections, such as tests of normality and adjustment for multiple comparisons
☐	☒	A full description of the statistical parameters including central tendency (e.g. means) or other basic estimates (e.g. regression coefficient) AND variation (e.g. standard deviation) or associated estimates of uncertainty (e.g. confidence intervals)
☐	☒	For null hypothesis testing, the test statistic (e.g. *F*, *t*, *r*) with confidence intervals, effect sizes, degrees of freedom and *P* value noted<br>*Give P values as exact values whenever suitable.*
☒	☐	For Bayesian analysis, information on the choice of priors and Markov chain Monte Carlo settings
☒	☐	For hierarchical and complex designs, identification of the appropriate level for tests and full reporting of outcomes
☒	☐	Estimates of effect sizes (e.g. Cohen's *d*, Pearson's *r*), indicating how they were calculated

Our web collection on statistics for biologists contains articles on many of the points above.

Software and code

Policy information about availability of computer code

Data collection	Thermofisher EPU used to collect data on microscope. SAXS experiments were performed at beamline 12-ID-B of the Advanced Photon Source (APS) at Argonne National Laboratory.
Data analysis	SIMPLE3.0 - available on GitHub https://github.com/hael/SIMPLE3.0; RELION3.0 - published and freely available; PHENIX - published and freely available; COOT - published and freely available; UCSF ChimeraX - published and freely available; PyMOL - published and freely available; ATSAS 2.8 - published and freely available; GNOM - published and freely available; SAXMOW - published and freely available; DAMMIF - published and freely available; DENSS - published and freely available; ICM - Molsoft, San Diego; BioPharma Finder 3.2 - Thermo Fisher Scientific, San Jose; FreeStyle software 1.7SP1 - Thermo Fisher Scientific, San Jose.

For manuscripts utilizing custom algorithms or software that are central to the research but not yet described in published literature, software must be made available to editors and reviewers. We strongly encourage code deposition in a community repository (e.g. GitHub). See the Nature Portfolio guidelines for submitting code & software for further information.

Data

Policy information about availability of data

All manuscripts must include a data availability statement. This statement should provide the following information, where applicable:

- Accession codes, unique identifiers, or web links for publicly available datasets
- A description of any restrictions on data availability
- For clinical datasets or third party data, please ensure that the statement adheres to our policy

All Supplementary Data files can be accessed at via Zenodo: https://doi.org/10.5281/zenodo.5514262
Supplementary Data 1 - Multiple sequence alignment for HTT used for Consurf analysis

Supplementary Data 2 - Multiple sequence alignment for HAP40 used for Consurf analysis
Supplementary Data 3 - Apo HTT cryo-EM map
Supplementary Data 4 - HTT-HAP40 Q23 regularised SAXS profile
Supplementary Data 5 - HTT-HAP40 Q54 regularised SAXS profile
Supplementary Data 6 - HTT-HAP40 Δexon 1 regularised SAXS profile
Supplementary Data 7 - XL-MS data
Supplementary Data 8 - HTT-HAP40 ensemble weightings
Supplementary Data 9 - HTT-HAP40 Q23 ensemble models
Supplementary Data 10 - HTT-HAP40 Q54 ensemble models
Supplementary Data 11 - HTT-HAP40 Δexon 1 ensemble models
Supplementary Video - Exon 1 of huntingtin (HTT) occupies different conformational space in the wildtype form of the HTT-HAP40 complex compared to Huntington's disease form

Raw and preprocessed mass spectrometry data used in this study is deposited in Figshare with identifier 839: (https://figshare.com/s/39b5b1a81838cd21ea92) and PRIDE through accession PXD028313. Also available through these links are CSMs tables that show the Scores and CSMs identified in our XLMS data sets as well as mass error.

Cryo-EM maps can be downloaded at EMDB 22106 and model coordinates at PDBID 6X9O.

All expression constructs are available through Addgene.

Field-specific reporting

Please select the one below that is the best fit for your research. If you are not sure, read the appropriate sections before making your selection.

- [x] Life sciences
- [ ] Behavioural & social sciences
- [ ] Ecological, evolutionary & environmental sciences

For a reference copy of the document with all sections, see nature.com/documents/nr-reporting-summary-flat.pdf

Life sciences study design

All studies must disclose on these points even when the disclosure is negative.

Sample size	Experiments were performed at least 2-3 times to confirm reproducibility. Sample sizes are described in Materials and Methods and are also detailed in figure legends.
Data exclusions	N/A
Replication	The data were generated from at least 3 technical replicates. In most cases the experiments were successfully repeated on separate dates. All details are indicated in the manuscript.
Randomization	N/A
Blinding	N/A

Reporting for specific materials, systems and methods

We require information from authors about some types of materials, experimental systems and methods used in many studies. Here, indicate whether each material, system or method listed is relevant to your study. If you are not sure if a list item applies to your research, read the appropriate section before selecting a response.

Materials & experimental systems

n/a	Involved in the study	
☐	☒	Antibodies
☐	☒	Eukaryotic cell lines
☒	☐	Palaeontology and archaeology
☐	☒	Animals and other organisms
☒	☐	Human research participants
☒	☐	Clinical data
☒	☐	Dual use research of concern

Methods

n/a	Involved in the study	
☒	☐	ChIP-seq
☒	☐	Flow cytometry
☒	☐	MRI-based neuroimaging

Antibodies

Antibodies used	HTT (Abcam EPR5526, Millipore MAB2166, Cell Signaling Technologies D7F7); HAP40 (Novus NBP2-54731, LSBio LS-C167891),

	anti-Flag (Sigma #F4799), vinculin (Abcam EPR8185), total protein (Licor 926-11010).
Validation	EPR5526 and MAB2166 are knock out validated for HTT as detailed by their respective manufacturers (Abcam and Millipore). D7F7 and #F4799 were only used on purified, recombinant flag-tagged HTT samples so did not require further validation. Vinculin and HAP40 antibodies were shown to produce a single band of the size corresponding to the expected mass of the protein in question.

Eukaryotic cell lines

Policy information about cell lines

Cell line source(s)	RPE1 (ATCC) CRL-4000
Authentication	Cell lines were not authenticated after purchase from supplier.
Mycoplasma contamination	Cells were negative for mycoplasma.
Commonly misidentified lines (See ICLAC register)	N/A

Animals and other organisms

Policy information about studies involving animals; ARRIVE guidelines recommended for reporting animal research

Laboratory animals	Species: Mus musculus. Strain: C57Bl/6J. Sex: Western blot all females; qPCR mixed male / female. Age: Western blot 5mo +/- a week; qPCR 5.5mo +/- two weeks
Wild animals	N/A
Field-collected samples	N/A
Ethics oversight	All procedures were reviewed and approved by the animal care and use committee at Western Washington University.

Note that full information on the approval of the study protocol must also be provided in the manuscript.
